# One in three reports pain in a given week: a one-season prospective study on prevalence of pain and analgesic use in amateur female and male football players

**DOI:** 10.1136/bmjsem-2025-002851

**Published:** 2026-01-03

**Authors:** Sofi Sonesson, Ida Åkerlund, Kalle Torvaldsson, Emmanuel Bäckryd, Hanna Lindblom, Martin Hägglund

**Affiliations:** 1 Department of Health, Medicine and Caring Sciences, Unit of Physiotherapy, and Sport Without Injury Programme (SWIPE), Linköping University, Linköping, Sweden; 2 Pain and Rehabilitation Center, and Department of Health, Medicine and Caring Sciences, Linköping University, Linköping, Sweden

**Keywords:** Sports medicine, Surveillance, Football, Female, Male

## Abstract

**Objectives:**

To study the prevalence of pain and analgesic use in amateur football players and explore sex-based and age-based differences.

**Methods:**

A prospective cohort study of 316 amateur football players (185 females, 131 males), mean age 20 years (range 15–54). Baseline data on demographics and pain history in the preceding season were collected. Players reported training/match participation, pain, analgesic use and injuries every 2 weeks over a 7-month season (April–October 2023).

**Results:**

2439 weekly reports were analysed. Weekly pain prevalence was 40.7% (95% CI 36.4% to 45.4%) in female players and 37.2% (32.4% to 42.7%) in male players. Moderate to severe pain was more frequently reported in youth females than youth males (weekly prevalence 20.5% (15.8% to 26.8%) vs 13.1% (9.6% to 17.9%), p=0.032). Female players reported more analgesic use than male players (27.6% (23.8% to 32.1%) vs 11.2% (8.4% to 14.8%), p<0.001). Gradual-onset injuries were the predominant cause of pain (47% in females, 50% in males). Over-the-counter paracetamol (female 70%, male 61%) and non-steroidal anti-inflammatory drugs (female 67%, male 44%) were most commonly used. Analgesic use was primarily driven by non-injury-related pain in females and gradual-onset injuries in males.

**Conclusions:**

Pain was common among amateur football players, with more than one-third of players reporting pain in a given week. Female youth players reported higher prevalence of moderate to severe pain, and both female youths and female adults used more analgesics than male counterparts. These findings call for sex-specific pain strategies and educational programmes targeting pain and medication management in amateur sports.

**WHAT IS ALREADY KNOWN ON THIS TOPIC**

- There are limited data on pain prevalence and analgesic use in amateur football players, especially data including sex-specific comparisons.
- Prospective data collection of analgesic use is needed to understand consumption patterns.

**WHAT THIS STUDY ADDS**

- This study reveals that pain is highly prevalent among amateur football players, with 40.7% of females and 37.2% of males reporting pain in a given week. Youth females experienced higher rates of moderate to severe pain compared with youth males (20.5% vs 13.1%). Female players reported more frequent analgesic use than male players (27.6% vs 11.2%).
- Analgesic use is widespread, particularly among female players, highlighting the need to address potential health risks related to pain-masking. The study shows that gradual-onset injuries are the leading cause of pain, but non-injury-related pain is a key driver of analgesic use in females.

**HOW THIS STUDY MIGHT AFFECT RESEARCH, PRACTICE OR POLICY**

- This study highlights the need for sex-specific and age-specific strategies for pain prevention and management, along with guidelines for responsible analgesic use in amateur football.
- It suggests the importance of monitoring analgesic use, especially among female athletes.
- The findings show the necessity of future research on the potential long-term health consequences of playing with pain or while taking medication.

## Introduction

 Pain is common in sports, resulting from, eg, acute injuries, overuse or chronic conditions. The causes of sport-related pain are multifactorial, including biomechanical stress, nociceptive gain and psychosocial factors.^1 2^ In a previous study evaluating pain associated with football-related injuries in amateur football players, we found that the weekly pain prevalence during activities of daily living averaged 17.2%, while the weekly pain prevalence during football was 18.3%, indicating a significant impact on both daily life and sport.^3^ However, pain perceptions are influenced by multiple factors, and pain can manifest despite the absence of objective tissue damage.^2^ While the previous study focused solely on football injury-related pain,^3^ comprehensive data on all types of pain during or related to sports—including pain occurring in the absence of injury—is lacking. This study addresses this gap by capturing a broader spectrum of pain experiences among amateur football players.

Analgesic use is widespread in elite sports,^4^,^6^ yet there is a notable lack of research on its prevalence and patterns in amateur sports settings.^6^ Amateur athletes might differ in pain perception or behaviour compared with elite counterparts due to differing prerequisites such as training load, access to medical support and experience with pain management strategies. Non-steroidal anti-inflammatory drugs (NSAIDs) are the most common analgesics in elite^7^ and youth^8^ athletes. In football World Cups between 2002 and 2014, the use of any medication was reported by 72% of adult female players, 69% of adult male players, and 60% of U-17 and U-20 male players, while NSAID use was reported by 51%, 55% and 43% of these groups, respectively.^9^ Although the 2018 World Cup saw a reduction in medication, 54% used any medication and 40% used NSAID during the tournament.^10^ Given that long-term use of NSAIDs may imply a risk of adverse effects,^5 8 11^ prospective medication data are needed.

A survey study in amateur football where coaches reported on their players’ analgesic use found that 36.2% of players used analgesics.^12^ However, a recent interview study with amateur football coaches revealed uncertainty about players’ actual consumption.^13^ This highlights the need for prospective data directly from players to accurately capture analgesic use during the season. Understanding the prevalence of pain and analgesic use during the season among amateur football players is crucial to inform effective pain prevention and management strategies. To address current knowledge gaps, we aimed to study the prevalence of pain and analgesic use in amateur female and male football players and to explore sex-based and age-based differences.

## Methods

### Study design

This prospective cohort study followed the Strengthening the Reporting of Observational Studies in Epidemiology (STROBE)^14^ and STROBE-Sport Injury and Illness Surveillance^15^ checklists.

### Setting

The study was conducted during the 2023 football season (April–October) in three football districts in south-central Sweden.

### Participants

Eligible participants were female and male players aged ≥15 years, playing in adolescent (16–19 years) or adult amateur leagues (male 4th–9th leagues (out of 9 leagues), female 3rd–6th leagues (out of 6 leagues)). Players were included regardless of health status at inclusion, except those with long-term injuries who did not plan to participate during the season.

Cluster sampling was conducted at the team level. Coaches of all 546 eligible teams were contacted via email. The emails were followed by telephone contact, through which coaches received oral and written information about the study. Coaches who agreed to participate forwarded information to their players and informed consent was obtained digitally by the players. Among the eligible teams, 60 teams agreed to participate in the study, 86 teams declined, and 400 did not respond to the invitation (online supplemental material 1).

### Variables

Key variables included pain experiences and analgesic use. Operational definitions are shown in table 1.

**Table 1:** Definitions and explanation of outcome variables

Pain	Experience of pain at any time during day or night, including all types of pain irrespective of the presence of sport injury or not
Moderate to severe pain	The extent of pain reported according to OSTRC-O2: no pain, mild pain, moderate pain, severe pain^16^
Playing football with pain	Experience of pain during football training or match play
Injury	Any physical complaint, regardless of the need for medical attention or time loss, including both sudden-onset and gradual-onset injuries^35^
Estimated weekly prevalence of pain and analgesic use	Calculated using the number of weekly reports indicating pain/analgesic use as the numerator, and the total number of weekly reports as the denominator
Estimated weekly prevalence of playing football with pain and analgesic use	Calculated using the number of weekly reports indicating playing football with pain/analgesic use as the numerator, and the number of weekly reports involving football play as the denominator

OSTRC-O2, Oslo Sports Trauma Research Center Overuse Injury Questionnaire, updated version.

### Data sources/measurement

A baseline survey was administered at the beginning of the football season (March–May). Throughout the 2023 season (April–October), players responded to a survey every second week pertaining to the previous 7 days. Biweekly reporting was chosen to reduce response burden and support consistent participation. Data were collected using web-based surveys (esMakerNX3 V.3.0) and were distributed via a link that was sent by email and/or short message service to the included players.

The baseline survey gathered data on basic demographics, general health and physical activity, and sport participation at the time of the survey, and pain experiences and analgesic use during football in the preceding 2022 season.

The prospective survey included questions about football training and match participation, experiences of pain during training and/or match play and during daily activities outside of football, and use of analgesics, in the preceding 7 days. In addition, injury occurrence was reported using the updated Oslo Sports Trauma Research Centre questionnaire on health problems.^16^ Players could select multiple response options within a single weekly report, such as reporting several pain locations, causes of pain, reasons for analgesic use and types of analgesics.

### Bias

To reduce non-response bias, reminders were sent to players via the survey system and coaches were encouraged to remind players in conjunction with team training. Up to two reminders were sent per weekly survey, and after that, non-responders were contacted over telephone to collect their answers.

### Study size

The final sample included players from 60 teams.

### Quantitative variables

Weekly prevalence of pain, moderate to severe pain and analgesic use were the primary variables. These were calculated for the total cohort and subgroups based on age (youths: 15–17 years and adults: ≥18 years) stratified by sex and presented with 95% CIs.

### Statistical analyses

All statistical analyses were conducted using IBM SPSS Statistics for Windows (V.29.0.2.0) and R Statistical Software (V.4.5.1; R Core Team 2025). An experienced statistician prepared the database and analysed the data.

An a priori sample size calculation for sex-comparison of the primary outcome—weekly pain prevalence—indicated that 270 players (135 females and 135 males) were needed to achieve at least 80% power (α=0.05), accounting for a 30% dropout rate. The calculation was based on estimated weekly pain prevalence rates of 24% in female^3^ and 19% in male^3^ players during the season.

Baseline data are presented descriptively. The response rate for the weekly survey was calculated as the number of responses to the survey divided by the number of distributed surveys.

To evaluate the association between sex and weekly prevalence of pain or analgesic use among all players, as well as within the subgroups of young and adult players, we fitted a marginal generalised estimating equations (GEE) model with a Poisson distribution and a log link function. The model specification was: *Outcome_ij_=exp (β_0_+β_1_ Sex_ij_),* where *Outcome_ij_* denotes the binary indicator of whether player *_i_* reported pain (or analgesic use) in week *_j_*j (1=yes, 0=no). The variable *Sex* was coded as a binary predictor. Within-player correlations across repeated weekly reports were accounted for by specifying *PlayerID* as the clustering variable and assuming an exchangeable working correlation structure. The GEE was fitted using the geeglm() function from the geepack package in R (V.1.3.13).

A sensitivity analysis was conducted restricted to players who completed at least 60% of their weekly reports.

A p<0.05 was considered significant. Data imputation was not performed.

### Patient and public involvement 

While patients or members of the public were not directly involved in the design or conduct of this study, the use of established tools grounded in community-based research helped ensure that the content was appropriate and meaningful for participants.

### Equity, diversity and inclusion statement

This study included both female and male football players from different socioeconomic backgrounds. The authorship team comprised a gender-balanced group of junior, mid-career and senior researchers from multiple academic disciplines.

## Results

### Participants

A total of 316 players (185 females and 131 males) completed at least 1 weekly survey and were included in the analyses (table 2). The average weekly response rate was 62.1% (95% CI 57.2% to 67.0%) among female players and 51.2% (95% CI 45.9% to 56.5%) among male players (on average, 8.3 and 6.9 weekly reports, respectively).

**Table 2:** Player characteristics at baseline and previous season

	Total		Youth players		Adult players	
	Female	Male	Female	Male	Female	Male
	(n=185)	(n=131)	(n=74)	(n=65)	(n=111)	(n=66)
Age (years), mean (95% CI)	21 (20–22)	20 (19–21)	16 (16–17)	16 (16–17)	24 (22–25)	23 (21–24)
Years playing football						
<5 years	12 (6.5%)	3 (2.3%)	8 (10.8%)	2 (3.1%)	4 (3.6%)	1 (1.5%)
5–10 years	77 (41.6%)	55 (42.0%)	55 (74.3%)	41 (63.1%)	22 (19.8%)	14 (21.2%)
11–15 years	63 (34.1%)	48 (36.6%)	11 (14.9%)	22 (33.8%)	52 (46.8%)	26 (39.4%)
>15 years	33 (17.8%)	25 (19.1%)	0 (–)	0 (–)	33 (29.7%)	25 (37.9%)
Weekly football training frequency*						
1–2 sessions	62 (33.5%)	27 (20.6%)	15 (20.3%)	9 (13.8%)	47 (42.3%)	18 (27.3%)
3 sessions	68 (36.8%)	45 (34.4%)	27 (36.5%)	13 (20.0%)	41 (36.9%)	32 (48.5%)
≥4 sessions	55 (29.7%)	59 (45.0%)	32 (43.2%)	43 (66.2%)	23 (20.7%)	16 (24.2%)
Weekly strength and conditioning training frequency*						
0 sessions	22 (11.9%)	14 (10.7%)	11 (14.9%)	1 (1.5%)	11 (9.9%)	13 (19.7%)
1–2 sessions	132 (71.4%)	71 (54.2%)	55 (74.3%)	40 (61.5%)	77 (69.4%)	31 (47.0%)
3 sessions	20 (10.8%)	20 (15.3%)	7 (9.5%)	10 (15.4%)	13 (11.7%)	10 (15.2%)
≥4 sessions	11 (5.9%)	26 (19.8%)	1 (1.4%)	14 (21.5%)	10 (9.0%)	12 (18.2%)
Other team match participation	36 (19.5%)	19 (14.5%)	27 (36.5%)	13 (20.0%)	9 (8.1%)	6 (9.1%)
Football profile school enrolment	37 (20.0%)	50 (38.2%)	17 (23.0%)	32 (49.2%)	20 (18.0%)	18 (27.3%)
Participation in other sport*	23 (12.4%)	14 (10.7%)	13 (17.6%)	6 (9.2%)	10 (9.0%)	8 (12.1%)
Current health perception						
Very good	84 (45.4%)	85 (64.9%)	34 (45.9%)	48 (73.8%)	50 (45.0%)	37 (56.1%)
Good	91 (49.2%)	43 (32.8%)	36 (48.6%)	16 (24.6%)	55 (49.5%)	27 (40.9%)
Fair	10 (5.4%)	3 (2.3%)	4 (5.4%)	1 (1.5%)	6 (5.4%)	2 (3.0%)
Poor	0 (–)	0 (–)	0 (–)	0 (–)	0 (–)	0 (–)
Occurrence of pain during football play or absence from football due to pain previous season†	141 (76.2%)	101 (77.1%)	55 (74.3%)	43 (66.2%)	86 (77.5%)	58 (87.9%)
Frequency of pain during football play previous season†						
Never	44 (23.8%)	30 (22.9%)	19 (25.7%)	22 (33.8%)	25 (22.5%)	8 (12.1%)
Rarely	53 (28.6%)	53 (40.5%)	23 (31.1%)	22 (33.8%)	30 (27.0%)	31 (47.0%)
Monthly	44 (23.8%)	32 (24.4%)	15 (20.3%)	14 (21.5%)	29 (26.1%)	18 (27.3%)
Weekly or more often	44 (23.8%)	16 (12.2%)	17 (23.0%)	7 (10.8%)	27 (24.3%)	9 (13.6%)
Occurrence of analgesic use during football play previous season†	79 (42.7%)	32 (24.4%)	31 (41.9%)	12 (18.5%)	48 (43.2%)	20 (30.3%)
Frequency of analgesic use during football play previous season†						
Never	106 (58.6%)	99 (76.2%)	43 (59.7%)	53 (82.8%)	63 (57.8%)	46 (69.7%)
Rarely	46 (25.4%)	26 (20.0%)	15 (20.8%)	10 (15.6%)	31 (28.4%)	16 (24.2%)
Monthly	29 (16.0%)	5 (3.8%)	14 (19.4%)	1 (1.6%)	15 (13.8%)	4 (6.1%)
Weekly or more often	0 (–)	0 (–)	0 (–)	0 (–)	0 (–)	0 (–)

All results are presented as frequencies and percentages if not otherwise mentioned.

* During the football season.

† Retrospective recall from the preceding season. Youths 15–17 years; adults ≥18 years.

### Descriptive data

In the baseline survey, three out of four players (females 76.2%, males 77.1%, p=0.893) reported pain during football play or absence from football due to pain in the preceding season. Female players more often reported having weekly or more frequent pain occurrences compared with male players (23.8% vs 12.2%, p=0.034). Also, more females reported analgesic use during football play (42.7% vs 24.4%, p<0.001) (table 2). Perceived causes of pain during football play were gradual-onset football injury (females 44.9%, males 49.6%), sudden-onset football injury (females 40.5%, males 43.5%), sudden-onset injury outside football (females 11.4%, males 3.8%), illness (females 5.9%, males 5.3%) and other causes; that is, headache, stomach-ache or menstrual pain, (females 15.1%, males 6.1%) (online supplemental Material 2).

### Outcome data

In total, 2439 completed weekly player reports were collected, of which 1090 indicated that the player experienced pain and/or used analgesics during that specific week (906 reported pain, 484 reported analgesic use, and 300 reported pain and analgesic use).

## Main results

### Prevalence of pain and analgesic use

During the 2023 season, the weekly prevalence of pain was 40.7% (95% CI 36.4% to 45.4%) among female players, and 37.2% (95% CI 32.4% to 42.7%) among male players (p=0.324). Youth females reported a higher prevalence of moderate to severe pain than youth males (20.5% (95% CI 15.8% to 26.8%) vs 13.1% (95% CI 9.6% to 17.9%), p=0.032). Weekly analgesic use was more prevalent among female players (27.5% (95% CI 23.8% to 32.1%) vs 11.2% (95% CI 8.4% to 14.8%), p<0.001). Similarly, females more often reported using analgesics in weeks playing football (weekly prevalence 26.4% (95% CI 22.3% to 31.3%) vs 9.1% (95% CI 6.6% to 12.7%), p<0.001) (table 3).

**Table 3:** Weekly prevalence and PR between females and males

		Total		Youth players		Adult players	
		(Female n=185, Male n=131)		(Female n=74, Male n=65)		(Female n=111, Male n=66)	
		Prevalence (95% CI); p value	Weekly reports	Prevalence (95% CI); p value	Weekly reports	Prevalence (95% CI); p value	Weekly reports
Primary outcomes							
Weeks with pain*	Female	40.7 (36.4 to 45.4)	1538	38.3 (32.2 to 45.4)	580	42.3 (36.7 to 48.7)	958
	Male	37.2 (32.4 to 42.7)	901	30.5 (24.4 to 38.0)	450	44.1 (37.2 to 52.3)	451
	PR	1.09 (0.92 to 1.30); 0.324		1.26 (0.95 to 1.66); 0.109		0.96 (0.77 to 1.20); 0.708	
Weeks with moderate or severe pain*	Female	21.1 (18.0 to 24.6)	1537	20.5 (15.8 to 26.8)	580	21.3 (17.6 to 25.7)	957
	Male	16.4 (13.4 to 20.1)	901	13.1 (9.6 to 17.9)	450	19.8 (15.3 to 25.5)	451
	PR	1.28 (0.99 to 1.65); 0.055		1.56 (1.04 to 2.36); 0.032		1.08 (0.78 to 1.48); 0.651	
Weeks with analgesic use*	Female	27.6 (23.8 to 32.1)	1534	29.2 (23.0 to 37.0)	579	26.6 (21.8 to 32.3)	955
	Male	11.2 (8.4 to 14.8)	899	7.9 (5.3 to 11.7)	449	14.6 (10.1 to 21.1)	450
	PR	2.47 (1.79 to 3.40); <0.001		3.72 (2.33 to 5.92); <0.001		1.82 (1.20 to 2.77); 0.005	
Secondary outcomes							
Weeks playing football with pain†	Female	42.8 (38.3 to 47.8)	1102	39.8 (33.2 to 47.6)	475	44.8 (39.0 to 51.5)	627
	Male	34.7 (29.8 to 40.4)	739	28.3 (22.1 to 36.3)	384	41.3 (34.4 to 49.6)	355
	PR	1.23 (1.02 to 1.49); 0.028		1.40 (1.03 to 1.91); 0.030		1.08 (0.86 to 1.37); 0.487	
Weeks playing football with moderate or severe pain†	Female	19.5 (16.4 to 23.3)	1102	18.0 (13.3 to 24.2)	475	20.5 (16.5 to 25.5)	627
	Male	14.8 (12.1 to 18.2)	739	12.1 (8.6 to 16.9)	384	17.7 (13.8 to 22.7)	355
	PR	1.32 (1.01 to 1.73); 0.045		1.49 (0.95 to 2.34); 0.082		1.16 (0.83 to 1.61); 0.379	
Weeks with pain outside football*	Female	36.5 (32.3 to 41.2)	1537	34.4 (28.6 to 41.5)	580	37.8 (32.3 to 44.3)	957
	Male	31.9 (27.3 to 37.3)	901	25.8 (20.0 to 33.3)	450	38.1 (31.4 to 46.2)	451
	PR	1.14 (0.94 to 1.39); 0.187		1.33 (0.97 to 1.83); 0.074		0.99 (0.77 to 1.27); 0.954	
Weeks with moderate or severe pain outside football*	Female	14.1 (10.1 to 19.6)	1537	15.4 (12.2 to 19.5)	580	27.6 (23.8 to 32.1)	957
	Male	6.9 (4.5 to 10.5)	901	10.9 (7.3 to 16.1)	450	11.2 (8.4 to 14.8)	451
	PR	2.04 (1.19 to 3.50); 0.010		1.42 (0.90 to 2.25); 0.134		2.47 (1.79 to 3.40); <0.001	
Weeks with analgesic use and football play†	Female	26.4 (22.3 to 31.3)	1099	27.9 (21.5 to 36.2)	474	25.3 (20.2 to 31.7)	625
	Male	9.1 (6.6 to 12.7)	737	6.3 (4.0 to 9.9)	383	12.1 (7.8 to 18.9)	354
	PR	2.90 (2.00 to 4.20); <0.001		4.41 (2.62 to 7.40); <0.001		2.09 (1.27 to 3.43); 0.004	

Youths 15 to 17 years; adults ≥18 years.

* All weekly reports included in analysis.

† Only weekly reports playing football included in analysis.

PR, prevalence ratio.

**Figure 1:**
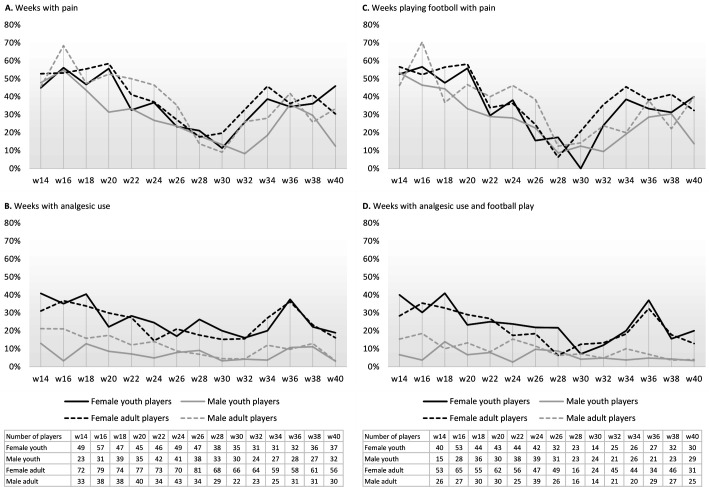
Seasonal trends in pain and analgesic-related behaviours among football players (prospective data). Prevalence of (A) pain, (**B**) analgesic use, (**C**) playing with pain and (D) weeks with analgesic use and football play. The number of players represents the number of respondents in each cohort for each calendar week. Calendar weeks 28 and 30 correspond to the summer break, during which many teams did not have any scheduled training sessions. Youths aged 15–17 years; adults ≥18 years.

Analgesic use was consistently higher among female players compared with male players throughout the season. In both groups, the prevalence of pain peaked at the beginning of the season, notably within the first 7 weeks. Seasonal trends in pain prevalence, playing with pain, analgesic use and analgesic use in weeks playing football are illustrated in figure 1.

### Pain locations and causes

**Figure 2:**
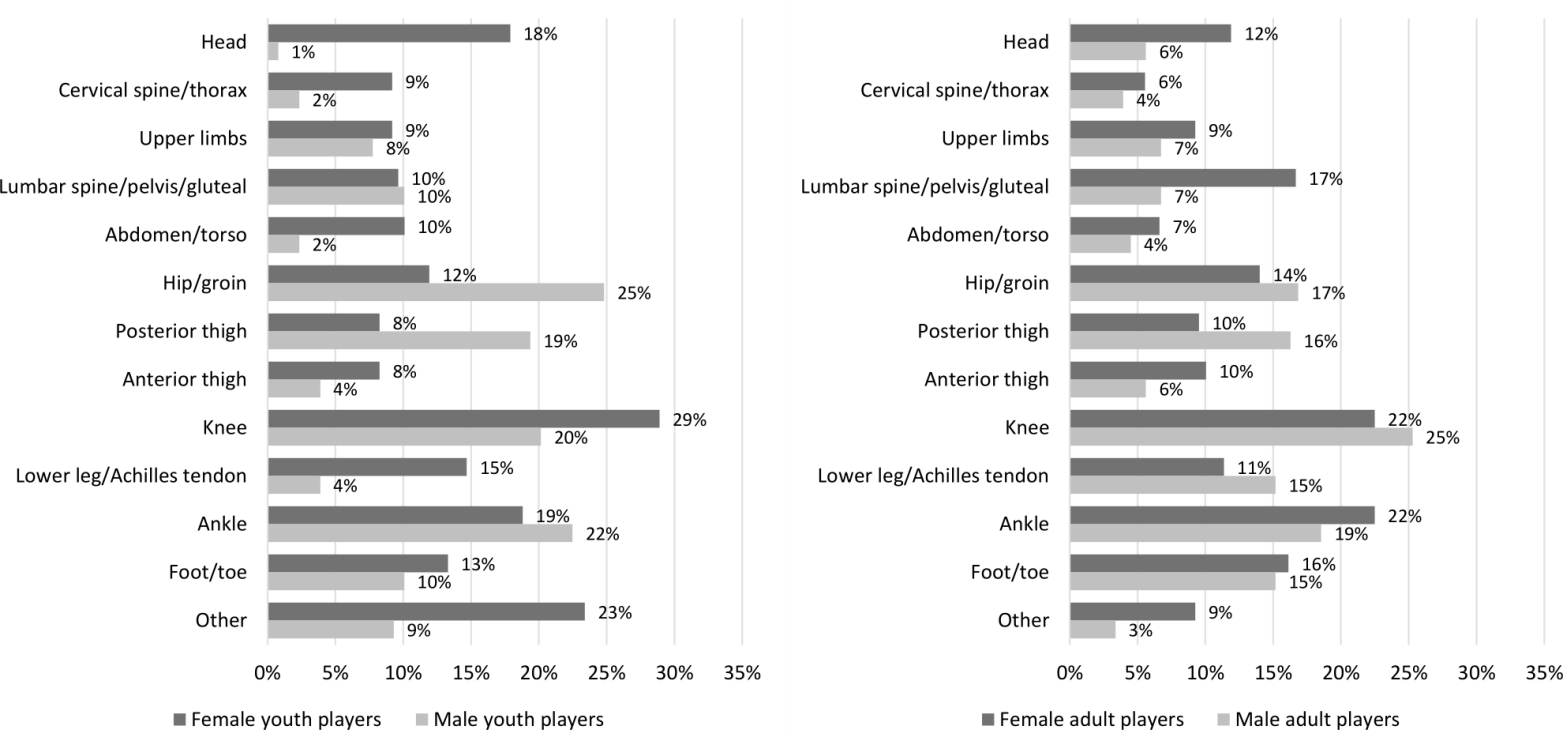
Pain locations for female and male youth and adult players. Calculations of pain locations (%) are based on all weekly reports where players reported pain (903 reports; 218 from female youths, 129 from male youths, 378 from female adults and 178 from male adults). Players could report more than one pain location. Youths aged 15–17 years; adults ≥18 years.

**Figure 3:**
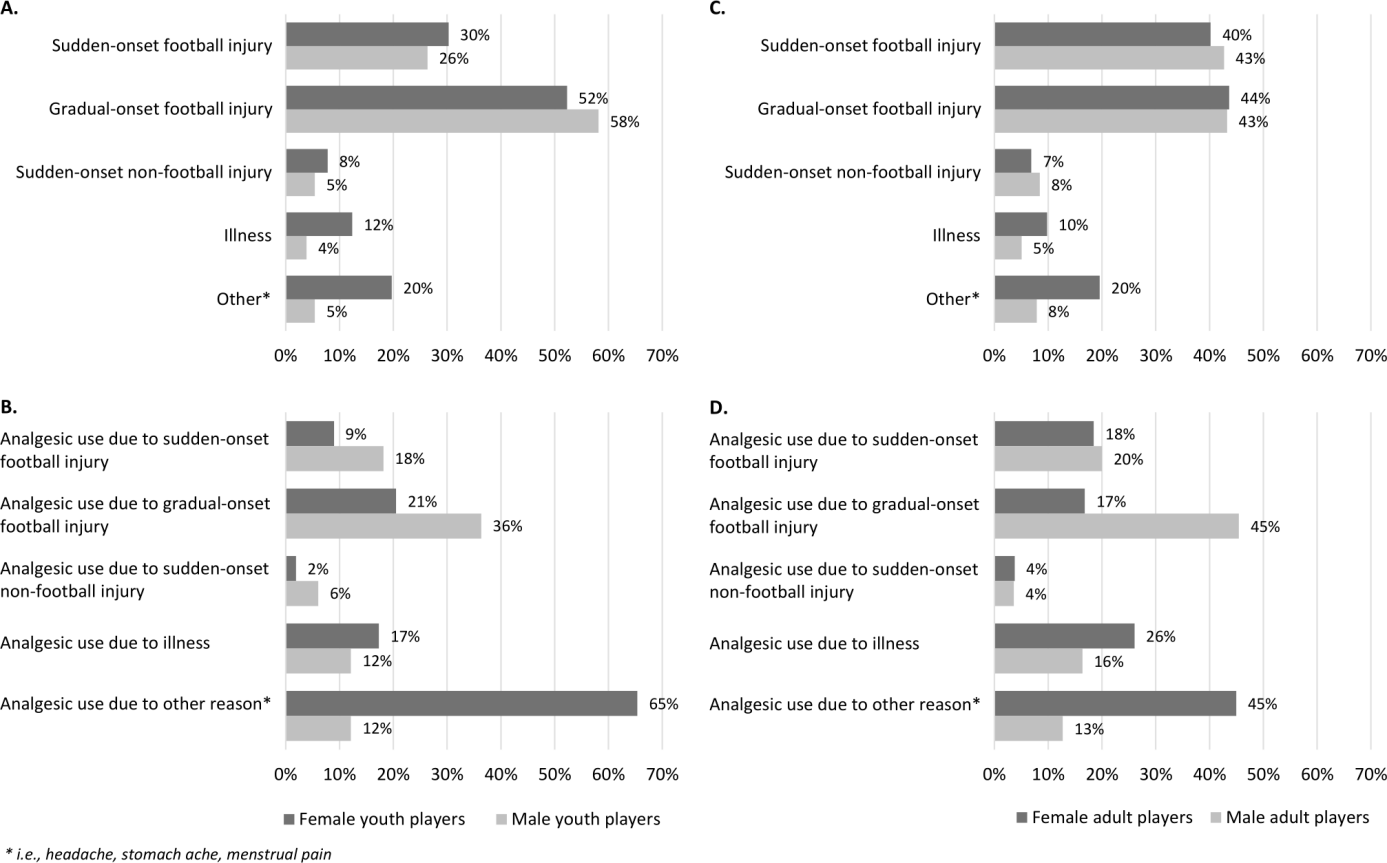
Causes of pain and reasons for analgesic use for female and male (A, B) youth and (C, D) adult players. Calculations of causes of pain (%) are based on all weekly reports where players reported pain (903 reports; 218 from female youths, 129 from male youths, 378 from female adults and 178 from male adults). Calculations of reasons for analgesic use (%) are based on all weekly reports where players reported analgesic use (482 reports; 156 from female youths, 33 from male youths, 238 from female adults and 55 from male adults). Players could report more than one cause of pain and more than one reason for analgesic use. Youths 15–17 years; adults ≥18 years.

Pain locations varied by age and sex. Among youth female players, the most commonly reported pain sites were the knee, ankle and head. Youth male players most frequently reported pain in the hip/groin, ankle and knee. Among adult players, in both females and males, pain was most commonly located in the knee and ankle, followed by lumbar spine/pelvis/gluteal in females and hip/groin in males (figure 2). Gradual-onset football injury was the most frequently reported cause of pain among youth players, accounting for 52% of pain weeks in females and 58% in males. Among adult players, both sudden-onset and gradual-onset injuries were reported with similar frequency, each contributing to 40%–44% of pain cases. Pain not related to injury or illness—such as headaches, stomach-aches and menstrual pain—accounted for 20% of pain weeks in females and 5%–8% in males (figure 3).

### Analgesic use patterns

Among females, pain not related to injury or illness was the leading reason for analgesic use, accounting for 65% of use weeks in youths and 45% in adults. Specifically, menstrual pain contributed to 15% in youth and 13% in adult females. Among males, gradual-onset injury was the leading reason for analgesic use, reported in 36% of youth cases and 45% of adult cases (figure 3).

**Figure 4:**
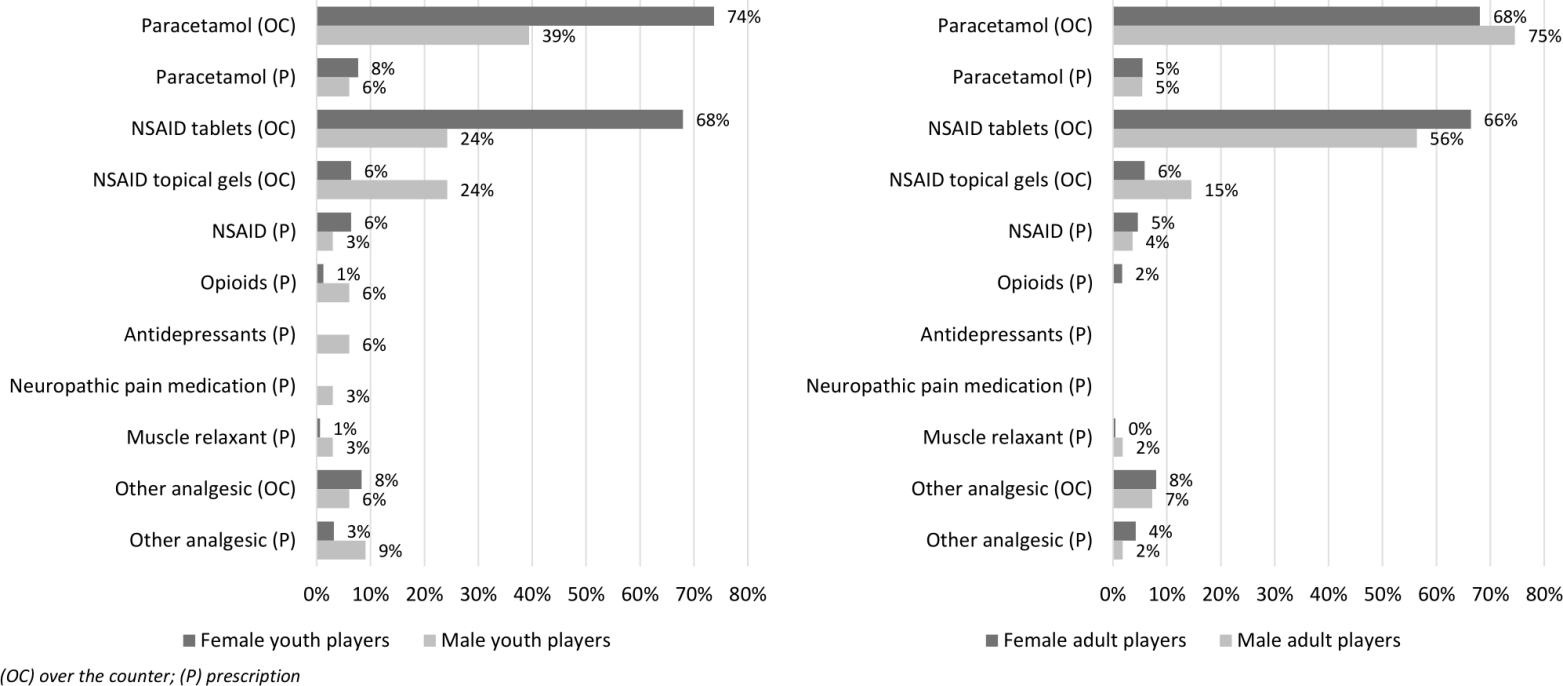
Types of analgesics (weeks with analgesic use) for female and male youth and adult players. Calculations of types of analgesics (%) are based on all weekly reports where players reported analgesic use (482 reports; 156 from female youths, 33 from male youths, 238 from female adults and 55 from male adults). Players could report more than one analgesic type. NSAID (**P**) includes both tablets and gels. Use of both Paracetamol and NSAID during the same week was reported by 56% of female youths, 18% of male youths, 48% of female adults and 42% of male adults. Youths 15–17 years; adults ≥18 years. NSAID, non-steroidal anti-inflammatory drug.

Over-the-counter paracetamol and NSAID tablets were the most commonly used analgesics. Among youth players, paracetamol was used in 74% of the weeks with analgesic use among females (115 weekly reports) and 39% among males (13 weekly reports). NSAIDs were used in 68% of those weeks in females (106 reports) and 24% in males (8 reports). Among adult players, paracetamol was used in 68% of analgesic-use weeks in females (162 reports) and 75% in males (41 reports). NSAID use was reported in 66% of those weeks among females (158 reports) and 56% among males (31 reports) (figure 4).

### Sensitivity analysis

Players who completed at least 60% of their weekly reports had similar prevalences of pain and analgesic use as reported in the main analysis (online supplemental Material 3).

## Discussion

This study demonstrates a substantial burden of pain among youth and adult amateur football players. Over one-third of all weekly reports included presence of pain, indicating that pain is frequent and ongoing throughout the season. Notably, moderate to severe pain was more commonly reported by youth female players, with a prevalence of 21% of weeks, compared with 13% among youth male players. Analgesic use was also more common among female players, who were more than twice as likely to use pain medication. Non-injury-related pain such as headaches, stomach-aches and menstrual pain was the leading reason for analgesic use among female players. The predominance of gradual-onset injuries as a reported cause of pain and reason for analgesic use in male players may indicate challenges in balancing load, recovery and overuse.^17^ Pain management in athletes requires balancing symptom relief with recognition of pain’s protective function in the presence of injury.^18^ Therefore, there is a need for proactive monitoring of pain symptoms, especially in female athletes, and the implementation of sex-specific and age-specific strategies for injury prevention, load management, pain management and education on safe analgesic use.

More than three out of four players reported experiencing pain during football play or absence from football due to pain in the preceding season, and nearly one in four female players reported weekly pain. The weekly pain prevalence, when capturing all types of pain during prospective registration, was 40.7% among female players and 37.2% among male players, which is twice as high as a similar study evaluating prevalence of pain associated with football-related injuries only (17.2%).^3^ The previous study was part of an injury prevention trial, which might lower the pain burden. Although part of the difference might be due to the prevalent reports of pain not related to sport, since the prevalence of chronic pain in young adults in the general population has been estimated to be 11.6%.^19^ Hence, in capturing all types of pain, the current study provides a more comprehensive picture of the burden of pain among amateur football players. Still, football injury is reported as a cause of pain in just over 80% of weeks with pain. Sports-related pain exists on a spectrum from pain related to an injury to pain without a clear pathology, which influences clinical reasoning and treatment approaches.^2^ Rather than only focusing on pathophysiological mechanisms, pain needs to be viewed through a broader lens—that is, the biopsychosocial model of pain^20^—which embraces the complex interplay between biological, psychological and social factors in the perception and management of pain.^21^ Interpreting athletes’ pain perceptions requires consideration of multiple factors, including fear of pain, performance pressure, individual pain appraisal and coping strategies.^22^ Athletes frequently face the dilemma of playing through pain; balancing short-term benefits like social connection, fitness and mental resilience against risks such as injury aggravation, delayed recovery and long-term health consequences.^13 23^ Although athletes, particularly those in contact sports,^24^ generally have high pain tolerance,^25 26^ this can be reduced by fear of pain.^25 27 28^ Ignoring pain might help athletes continue to play despite pain, whereas coping styles like catastrophising may limit participation.^29^ Amateur football players have a high degree of pain acceptance that is strongly influenced by social and psychological factors,^23^ which partly could explain the age and sex differences in pain perceptions seen in the present study.

Most athletes reported only using over-the-counter analgesics, which is in line with previous data on youth elite athletes.^30^ Female players reported using analgesics in approximately one out of 4 weeks. Females were 2.7 times more likely than males to use analgesics in weeks playing football. Higher medication use among females has previously been reported in data from football World Cups^9^ as well as among youth elite athletes and students.^31^ More generally, it is well-established that women use analgesics more often than men,^32^ a fact that probably mirrors the higher prevalence of chronic pain conditions in women.^33^

The primary reason for analgesic use among female players was non-injury-related pain, where the prevalence of menstrual pain as a reason for analgesic use was 15% in youth and 13% in adult female players. In contrast, male players primarily used analgesics due to gradual-onset injury. It has been shown that analgesic use is prevalent both in youth athletes and the general youth population, with an average weekly prevalence of approximately 20%.^31^ Athletes often used analgesics to alleviate pain or injury prior to or after sports participation and to prevent pain during sports participation.^31^ Many athletes are willing to use analgesics to compete, especially when injuries threaten their performance or sporting success.^34^ This raises concerns about the normalisation of analgesic use in sports. Prolonged NSAID use poses risks to cardiovascular, gastrointestinal and renal systems and may impair musculoskeletal tissue healing and remodelling.^11^ Given these risks, there is a need for preventive interventions, including structured education on safe medication practices.

### Clinical implications

This study provides important insights for clinicians, coaches and sports organisations working with amateur football players. The high prevalence of pain and widespread use of over-the-counter analgesics—especially among female athletes—calls for proactive and informed approaches to pain management.

Key clinical and policy implications include:

**Risk of masking pain:** Routine use of analgesics to enable continued participation may delay diagnosis, hinder recovery and increase the risk of long-term musculoskeletal complications. Clinicians should assess not only the presence of pain but also the context and frequency of analgesic use.

**Nuanced pain management:** In some cases, such as menstrual pain, analgesic use may support athlete’s well-being and participation in sport. This underscores the need for individualised, sex-specific pain management strategies that balance performance goals with health protection.

**Collaborative decision-making:** Decisions about playing while in pain should involve athletes, coaches and healthcare professionals, ensuring a shared understanding of potential risks and long-term consequences.

**Education and guidelines:** Sports organisations should develop clear, evidence-based guidelines for pain management and analgesic use, tailored to different age and sex groups. Educational efforts targeting players, coaches and medical staff are essential to promote safe medication practices and encourage timely treatment and recovery. Integrating pain management into coach education may be feasible to promote a healthier and more informed approach within youth and amateur football.

### Methodological considerations

The main strengths of this study include the prospective design that captured data every 2 weeks throughout an entire season, complemented by retrospective reports from the previous season. It comprehensively captured all types of pain and analgesic use across female and male youth and adult players. Data collection was conducted using a biweekly reporting interval to reduce participant survey response burden; however, this approach may increase the risk of missing short-term symptoms and underestimate weekly prevalence. Additionally, retrospective baseline data may be subject to recall bias. All data were self-reported, introducing potential biases and uncertainty regarding pain causes. Limited data on dosing and timing restricts conclusions about sport-related analgesic behaviours, such as playing through pain. The questions regarding analgesic use were developed specifically for this study, informed by the multiprofessional expertise of the research team; however, the questionnaire has not undergone formal validation. The early-season peak in pain prevalence may stem from training load changes during the transition from pre-season to competition, underlying health issues or increased reporting enthusiasm at study onset. The sample size was determined a priori based on the primary outcome—weekly pain prevalence—with a target of 270 players equally distributed by sex. Consequently, subgroup analyses, particularly those involving smaller groups, should be interpreted with caution due to limited statistical power. Higher female response rate could bias the observed sex differences. Differences in sport schedules and healthcare access across countries may limit the generalisability of the findings.

## Conclusions

Pain was common among amateur football players, with more than one-third reporting pain in a given week. Female youth players experienced a higher prevalence of moderate to severe pain, and both female youths and female adults reported more frequent analgesic use than male counterparts. Gradual-onset injuries were the leading cause of pain, although non-injury-related pain was the primary driver of analgesic use in females. These findings call for targeted, sex-specific strategies to prevent and manage pain, and for the development of clear, evidence-based guidelines on analgesic use in amateur sports.

## Supplementary material

10.1136/bmjsem-2025-002851 online supplemental file 1

10.1136/bmjsem-2025-002851 online supplemental file 2

10.1136/bmjsem-2025-002851 online supplemental file 3

## Data Availability

Data are available on reasonable request.
