# Supplementary figures and images for: One in three reports pain in a given week: a one-season prospective study on prevalence of pain and analgesic use in amateur female and male football players
Source: BMJ Open Sport Exerc Med. 2026 Jan 3;12(1):e002851. doi: 10.1136/bmjsem-2025-002851 (PMC12766771; doi:10.1136/bmjsem-2025-002851)

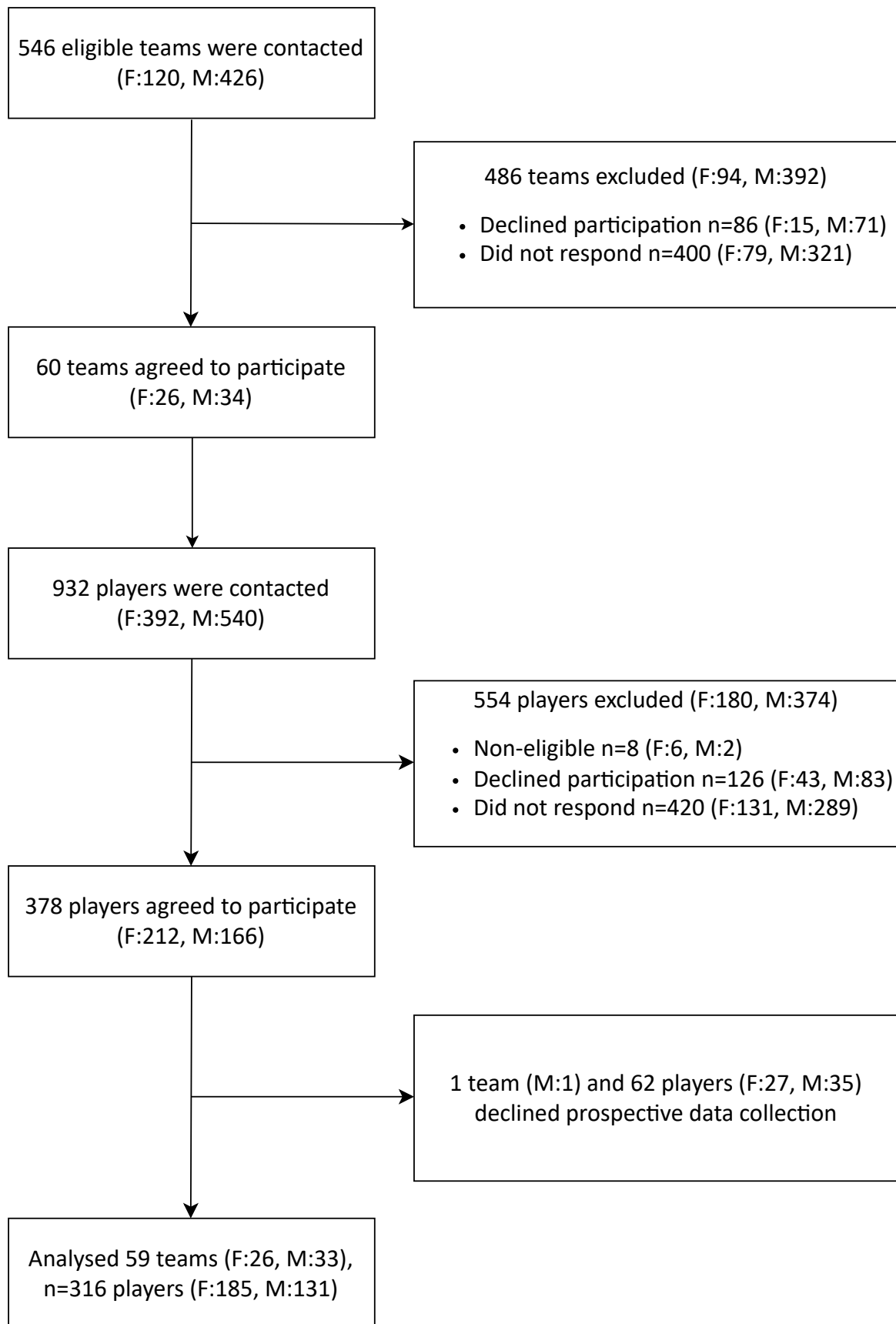

Supplement: online supplemental file 1 [file bmjsem-12-1-s001.pdf]
