# Supplementary material for: One in three reports pain in a given week: a one-season prospective study on prevalence of pain and analgesic use in amateur female and male football players
Source: BMJ Open Sport Exerc Med. 2026 Jan 3;12(1):e002851. doi: 10.1136/bmjsem-2025-002851 (PMC12766771; doi:10.1136/bmjsem-2025-002851)
Supplement: online supplemental file 2 [file bmjsem-12-1-s002.docx]

| Supplementary material 2. Pain prevalence in the preceding season | | | |  |  |  |
| --- | --- | --- | --- | --- | --- | --- |
|  | Total | | Youth players | | Adult players | |
|  | Female | Male | Female | Male | Female | Male |
|  | (n = 185) | (n = 131) | (n = 74) | (n = 65) | (n = 111) | (n = 66) |
| Pain in upper extremity/torso during football play | 69 (37.3%) | 44 (33.6%) | 25 (33.8%) | 18 (27.7%) | 44 (39.6%) | 26 (39.4%) |
| Pain in lower extremity during football play | 133 (71.9%) | 93 (71.0%) | 52 (70.3%) | 41 (63.1%) | 81 (73.0%) | 52 (78.8%) |
| Reason for pain during football play |  |  |  |  |  |  |
| Sudden-onset football injury | 75 (40.5%) | 57 (43.5%) | 24 (32.4%) | 21 (32.3%) | 51 (45.9%) | 36 (54.5%) |
| Gradual-onset football injury | 83 (44.9%) | 65 (49.6%) | 31 (41.9%) | 30 (46.2%) | 52 (46.8%) | 35 (53.0%) |
| Sudden-onset non-football injury | 21 (11.4%) | 5 (3.8%) | 9 (12.2%) | 2 (3.1%) | 12 (10.8%) | 3 (4.5%) |
| Illness | 11 (5.9%) | 7 (5.3%) | 4 (5.4%) | 3 (4.6%) | 7 (6.3%) | 4 (6.1%) |
| Other* | 28 (15.1%) | 8 (6.1%) | 11 (14.9%) | 5 (7.7%) | 17 (15.3%) | 3 (4.5%) |
| Chronic pain |  |  |  |  |  |  |
| No long-term pain | 87 (47.0%) | 60 (45.8%) | 34 (45.9%) | 33 (50.8%) | 53 (47.7%) | 27 (40.9%) |
| Up to 4 weeks | 28 (15.1%) | 37 (28.2%) | 16 (21.6%) | 19 (29.2%) | 12 (10.8%) | 18 (27.3%) |
| 1–3 months | 19 (10.3%) | 19 (14.5%) | 8 (10.8%) | 7 (10.8%) | 11 (9.9%) | 12 (18.2%) |
| 3–12 months | 27 (14.6%) | 9 (6.9%) | 8 (10.8%) | 5 (7.7%) | 19 (17.1%) | 4 (6.1%) |
| 1–3 years | 15 (8.1%) | 3 (2.3%) | 5 (6.8%) | 0 (–) | 10 (9.0%) | 3 (4.5%) |
| More than 3 years | 9 (4.9%) | 3 (2.3%) | 3 (4.1%) | 1 (1.5%) | 6 (5.4%) | 2 (3.0%) |
| All results are presented as frequencies and percentages | | | | | | |
| * i.e., headache, stomach-ache, menstrual pain. Youths 15–17 years; Adults ≥ 18 years. | | | | | | |
