# Supplementary material for: One in three reports pain in a given week: a one-season prospective study on prevalence of pain and analgesic use in amateur female and male football players
Source: BMJ Open Sport Exerc Med. 2026 Jan 3;12(1):e002851. doi: 10.1136/bmjsem-2025-002851 (PMC12766771; doi:10.1136/bmjsem-2025-002851)
Supplement: online supplemental file 3 [file bmjsem-12-1-s003.docx]

Supplementary material 3. Sensitivity analysis: Weekly prevalence and prevalence ratio between females and males on players with 8 or more weekly reports

|  | | Total | | Youth players | | Adult players | |
| --- | --- | --- | --- | --- | --- | --- | --- |
|  |  | (Female n=114, Male n=57) | | (Female n=41, Male n=28) | | (Female n=73, Male n=29) | |
|  |  | Prevalence  (95% CI); p-value | Weekly reports | Prevalence  (95% CI); p-value | Weekly reports | Prevalence  (95% CI); p-value | Weekly reports |
| **Primary outcomes** | | | | | | | |
| Weeks with pain^1^ | Female | 36.6 (31.9–42.0) | 1326 | 36.6 (29.7–45.0) | 480 | 36.6 (30.6–43.9) | 846 |
|  | Male | 31.7 (26.1–38.5) | 640 | 26.1 (19.0–36.1) | 315 | 37.1 (29.6–46.6) | 325 |
|  | PR | 1.15 (0.91–1.46); 0.233 |  | 1.40 (0.95–2.05); 0.087 |  | 0.99 (0.74–1.32); 0.930 |  |
| Weeks with moderate or severe pain^1^ | Female | 18.7 (15.5–22.6) | 1325 | 19.3 (13.8–27.1) | 480 | 18.3 (14.6–22.9) | 845 |
|  | Male | 13.5 (10.2–17.8) | 640 | 10.1 (6.8–15.1) | 315 | 16.6 (11.7–23.8) | 325 |
|  | PR | 1.39 (0.99–1.94); 0.055 |  | 1.90 (1.13–3.22); 0.016 |  | 1.10 (0.72–1.68); 0.655 |  |
| Weeks with analgesic use^1^ | Female | 23.6 (19.5–28.6) | 1323 | 25.8 (18.7–35.6) | 479 | 22.4 (17.7–28.3) | 844 |
|  | Male | 7.9 (5.5–11.2) | 638 | 5.5 (3.6–8.6) | 314 | 10.1 (6.3–16.1) | 324 |
|  | PR | 3.01 (2.02–4.48); <0.001 |  | 4.66 (2.70–8.04); <0.001 |  | 2.22 (1.31–3.76); 0.003 |  |
| **Secondary outcomes** | | | | | | | |
| Weeks playing football with pain^2^ | Female | 39.0 (34.1–44.8) | 939 | 38.6 (31.1–48.0) | 393 | 39.3 (33.0–46.8) | 546 |
|  | Male | 31.5 (25.7–38.5) | 525 | 26.1 (18.6–36.7) | 270 | 36.8 (29.0–46.6) | 255 |
|  | PR | 1.24 (0.97–1.58); 0.082 |  | 1.48 (0.99–2.21); 0.057 |  | 1.07 (0.80–1.44); 0.656 |  |
| Weeks playing football with moderate or severe pain^2^ | Female | 17.7 (14.3–22.0) | 939 | 17.0 (11.6–25.0) | 393 | 18.1 (14.0–23.4) | 546 |
|  | Male | 12.2 (9.4–15.8) | 525 | 9.5 (6.0–15.2) | 270 | 14.8 (11.1–19.8) | 255 |
|  | PR | 1.45 (1.03–2.03); 0.031 |  | 1.79 (0.98–3.27); 0.058 |  | 1.22 (0.83–1.80); 0.309 |  |
| Weeks with pain outside football^1^ | Female | 33.5 (28.9–38.9) | 1325 | 33.6 (26.9–42.1) | 480 | 33.4 (27.6–40.6) | 845 |
|  | Male | 26.6 (21.3–33.3) | 640 | 21.3 (14.4–31.4) | 315 | 31.8 (24.6–41.0) | 325 |
|  | PR | 1.26 (0.96–1.65); 0.091 |  | 1.58 (1.01–2.47); 0.046 |  | 1.05 (0.76–1.45); 0.756 |  |
| Weeks with moderate or severe pain outside football^1^ | Female | 13.2 (10.4–16.7) | 1325 | 13.0 (8.6–19.6) | 480 | 13.3 (10.0–17.7) | 845 |
|  | Male | 6.9 (4.4–10.9) | 640 | 4.7 (2.6–8.2) | 315 | 9.1 (4.9–16.8) | 325 |
|  | PR | 1.90 (1.14–3.19); 0.014 |  | 2.78 (1.38–5.61); 0.004 |  | 1.46 (0.74–2.88); 0.271 |  |
| Weeks with analgesic use and football play^2^ | Female | 23.5 (19.0–29.2) | 937 | 26.4 (18.8–37.0) | 392 | 21.7 (16.5–28.6) | 545 |
|  | Male | 6.7 (4.6–9.8) | 523 | 6.0 (3.8–9.5) | 269 | 7.5 (4.2–13.3) | 254 |
|  | PR | 3.50 (2.26–5.41); <0.001 |  | 4.41 (2.48–7.84); <0.001 |  | 2.89 (1.53–5.48); 0.001 |  |
| Youths 15–17 years; Adults ≥ 18 years; PR, prevalence ratio. | | | | | | | |
| ^1^ all weekly reports included in analysis; ^2^ only weekly reports playing football included in analysis | | | | | | | |
